# Relapsing MRI-negative myelitis associated with myelin-oligodendrocyte glycoprotein autoantibodies: a case report

**DOI:** 10.1186/s12883-022-02837-5

**Published:** 2022-08-24

**Authors:** Jan Kolcava, Aneta Rajdova, Eva Vlckova, Pavel Stourac, Josef Bednarik

**Affiliations:** 1 Faculty of Medicine, Masaryk University Brno, Brno, Czech Republic; 2 Department of Neurology, University Hospital Brno, Brno, Czech Republic; 3 ERN Neuromuscular Center: Euro-NMD, University Hospital Brno, Jihlavská 340/20, 625 00 Brno-Stary Liskovec, Brno-Bohunice, Czech Republic

**Keywords:** Demyelinating diseases, Myelin-oligodendrocyte glycoprotein, Evoked potentials, Magnetic resonance imaging, Case report

## Abstract

**Background:**

Serum antibodies to myelin-oligodendrocyte glycoprotein (MOG) are biomarkers of MOG-IgG-associated disorder (MOGAD), a demyelinating disease distinct from both multiple sclerosis and aquaporin-4-IgG neuromyelitis optica spectrum disorder. The phenotype of MOGAD is broad and includes optic neuritis, transverse myelitis, and acute demyelinating encephalomyelitis. Myelitis is common with MOGAD and typically results in acute and severe disability, although prospects for recovery are often favorable with prompt immunotherapy.

**Case presentation:**

This contribution presents a unique case report of a young male patient exhibiting relapsing myelitis with normal spinal cord and brain magnetic resonance imaging. Comprehensive diagnostic assessment revealed myelin-oligodendrocyte glycoprotein-IgG-associated disorder.

**Conclusion:**

MOGAD is one of the conditions which should be considered in MRI-negative myelitis. The diagnosis, however, may prove difficult, especially if the patient is not exhibiting other neurological symptoms of MOGAD. Conus or epiconus involvement is common in MOGAD; the patient reported herein exhibited incomplete rostral epiconus symptoms which, together with somatosensory evoked potential abnormalities, led to the diagnosis.

## Background

Serum antibodies to myelin-oligodendrocyte glycoprotein (MOG) are biomarkers of MOG-IgG-associated disorder (MOGAD), a demyelinating disease distinct from both multiple sclerosis (MS) and aquaporin-4-IgG neuromyelitis optica spectrum disorder (NMOSD). MOGAD phenotype include optic neuritis, myelitis, and attacks of brain or brainstem dysfunction (e.g., acute demyelinating encephalomyelitis, cortical encephalitis) [1]. Myelitis is common with MOGAD and typically results in acute and severe disability, although prospects for recovery are often favorable with prompt immunotherapy. Magnetic resonance imaging (MRI) of the spinal cord may show longitudinally extensive T2-hyperintense lesions (> 3 vertebral body segments), short lesions, or both, sometimes gray-matter-restricted in a linear configuration on sagittal images and/or “H”-shaped axially [2]. Diagnosis may be rendered difficult by heterogeneous clinical and radiographic findings.

## Case presentation

During the course of a respiratory infection, a 26-year old male exhibited gait imbalance, numbness and “pins and needles” the lower-limbs. Based on the patient´s description, the sensory symptoms were present in whole lower legs from the hips and proximal thigh distally (i.e. approximately in the distribution from L3 to S1). The patient suffered from cough and fever for 2 days prior the onset of neurological symptoms. His C-reactive protein (CRP) was 160 mg/L (normal range 0-5 mg/L). The chest X-ray, abdominal ultrasound and echocardiography were normal. The paranasal sinuses radiography revealed a mild gas-fluid level in the left maxillary sinus. Starting from the 4th day of the respiratory symptoms (2 day after the onset of neurological symptoms), the patient was treated with amoxicillin/clavulanic acid for seven days, first five days intravenously 1000 mg/200 mg every eight hours in the department of internal medicine of a small local hospital and two last days per orally 875 mg/125 mg twice a day with the good effect on respiratory functions and fever, but without any impact in neurological symptoms and signs. The neurological symptoms were, however, not dominant from the patient’s perspective and the patient was not examined by any neurologist at the beginning of the symptoms. The residual neurological symptoms (numbness in the lower legs and difficulties running and tandem-walking) remained stable for another three years. Since the level of disability was considered mild, the patient was still not referred to a neurologist. The patient’s family history was unremarkable, patient’s mother suffers from high blood pressure and type 2 diabetes mellitus, his father has high blood pressure. No neurological or autoimmune diseases appeared in the family. The patient suffered from mononucleosis at the age of ten and mumps at the age of 17, otherwise is the patient’s medical history unremarkable.

By the time the patient had reached the age of 29, a subacute progression (two weeks) of imbalance had resulted in severe walking problems and he was admitted to the neurological department of the secondary hospital. Neurological examination revealed perianogenital hypesthesia with sphincter dysfunction (urinary retention¨and incomplete empting) and severe positive and negative sensory symptoms in the lower limbs, including L3-S2 hypoesthesia, impaired joint position sense and loss of vibration sense, and severe lower limbs and gait ataxia. Deep-tendon reflexes and the plantar reflex were absent. The patient was unable to walk correctly in tandem gait, or to run due to ataxia, he was unable to walk unsupported for more than few meters and did not exhibit any muscle weakness. His EDSS was 6.0. The clinical findings in the cranial nerves and upper extremities were completely normal and his cognitive status was intact.

**Fig. 1:**
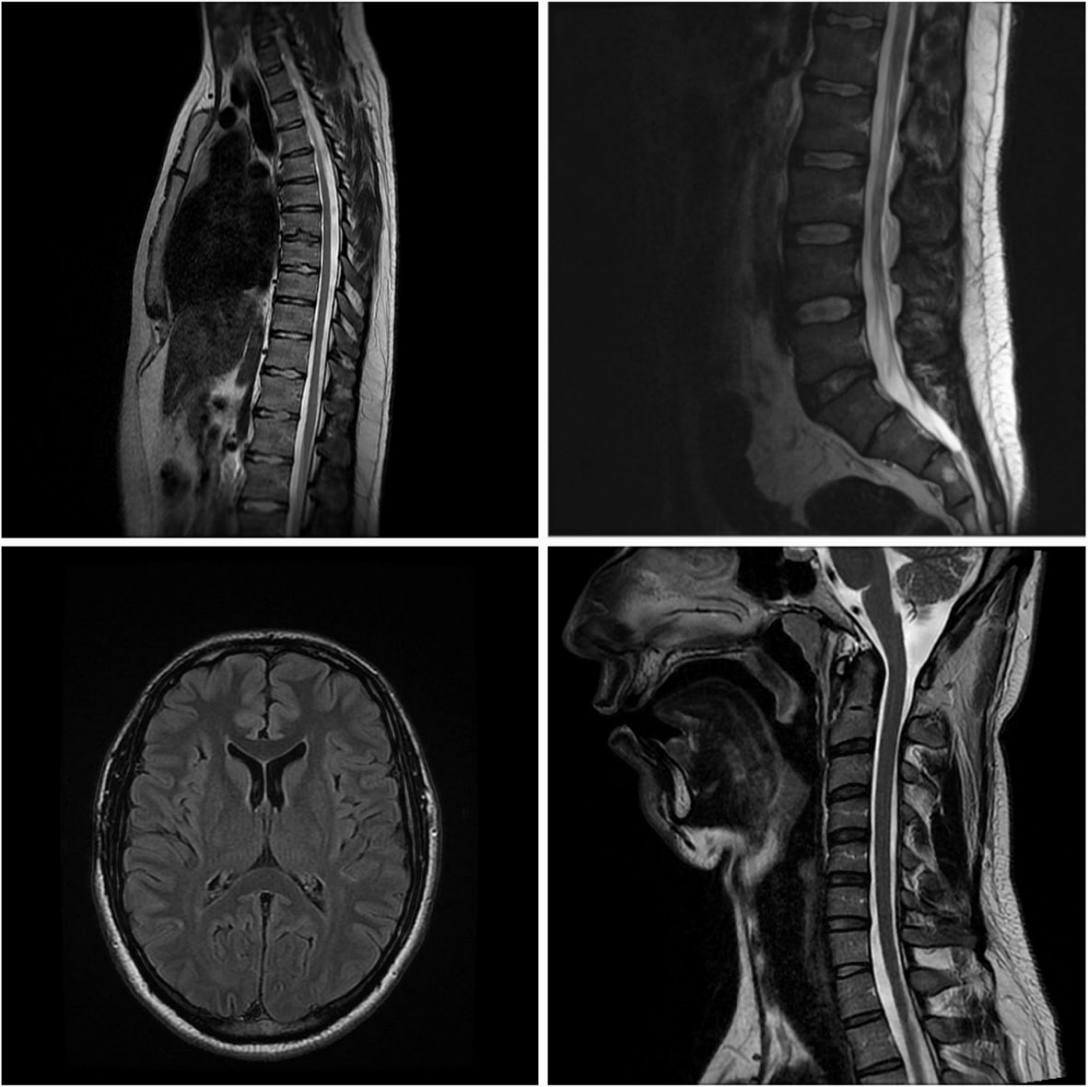
Magnetic resonance imaging scan of the brain and cervical, thoracic and lumbar spine (SIGNA Voyager 1.5T)

An MRI (1.5 T) scan of the brain and cervical, thoracic, and lumbar spine performed ten days after symptoms onset proved normal (Fig. 1). In the cerebrospinal fluid (CSF), there were no indications of neuro-infection, no oligoclonal bands appeared in CSF or in the serum. CSF protein was elevated to 2.24 g/L (normal range 0.15-0.45 g/L). Initial nerve conduction studies and needle electromyography (NCS/EMG) examinations were normal.

Based on the clinical presentation, relapsing-remitting course, elevated CSF protein levels and normal brain and spine MRI, a diagnosis of inflammatory polyneuropathy considered and the patient was therefore treated with high-dose steroids (3 g of methylprednisolone), resulting in a significant reduction of complaints with residual symptoms. Thus, the chronic treatment with prednisone (20 mg/day) commenced, and the patient was referred to a tertiary neuromuscular center.

**Fig. 2:**
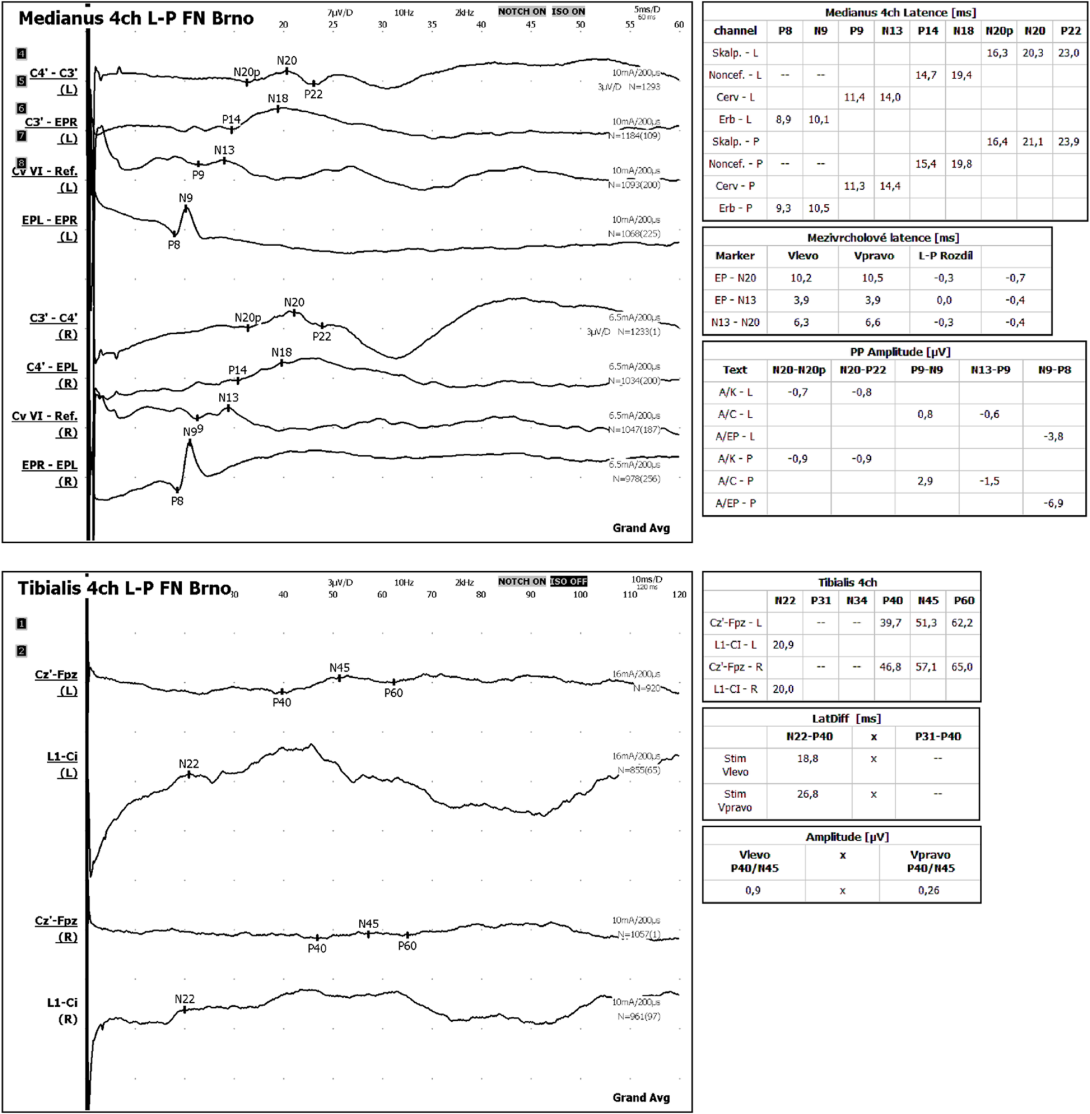
Somatosensory-evoked potentials established a central lesion of the spinal somatosensory pathway to the lower extremities

In this center, a follow-up NCS examination confirmed the absence of demyelinating changes in all the peripheral nerves examined in both upper and lower limbs. The spinal MRI did not show any abnormality of the lumbar or sacral nerve roots (i.e. no thickening or a tube-shaped enlargement). The diagnosis of inflammatory neuropathy including chronic immune sensory polyradiculopathy (CISP) was therefore considered improbable and further investigation undertaken [3, 4]. Visual, brainstem auditory and motor-evoked potentials were normal. Somatosensory-evoked potentials established a central lesion of the spinal somatosensory pathway to the lower extremities (with no abnormality in the upper extremities) (Fig. 2).

A range of laboratory tests was performed to investigate the following antibodies: the paraneoplastic (anti-Hu, anti-Ri, anti-Yo, anti-CV2, anti-Amphiphysin, anti-Ma1, anti-Ma2), the infectious (including syphilis and HIV) and the autoimmune (including anti-nuclear antibodies, antibodies against extractable nuclear antigens, anti-double- and single-stranded DNA; anti-neutrophil cytoplasmic antibodies, anticardiolipin antibodies, anti-cyclic citrullinated peptide antibodies and anti-aquaporin-4 (AQP4)), all proved negative. The blood levels of creatine kinase, myoglobin, vitamin B12, B9, Cu, thyroid-stimulating hormone, thyroxine and carbohydrate-deficient transferrin were normal. Eventually, anti-MOG antibodies were detected by immunofluorescence Euroimmun fixed cell-based assay (titer 1:80).

A diagnosis of MRI-negative MOGAD myelitis was established and, in response to persisting neurological symptoms, the patient was retreated with high-dose steroids (3 g of methylprednisolone), to no significant effect. Long-term treatment with 100 mg azathioprine was started (a reduced dose in the light of lower thiopurine methyltransferase activity). The previous treatment with 20 mg prednisone was progressively discontinued over the course of six months [5]. The medication is well tolerated; the patient had no complaints related specifically to the drug. The course of the disease was relapsing. There were no signs of relapse-independent progression.

Currently, the patient has been relapse-free and clinically stable for almost three years. MRIs of the brain and the spinal cord six months later remained normal. Anti-MOG seropositivity was repeatedly proven during remission by visual observation on a fluorescence microscope immunofluorescence Euroimmun fixed cell-based assay (titer 1:80 to 1:40). The patient’s optical coherence tomography remained normal.

At the age of 31, the patient currently exhibits mild negative sensory symptoms in the distal parts of the lower limbs, areflexia of the lower limbs, impaired sense of vibration and joint position, and moderate gait and lower limb ataxia. Tandem walking and running are not possible. His expanded disability status scale stands at 3.5 (cerebellar and sensitive 3, other functional systems normal). Patient´s cognitive functions are within normal range.

## Discussion and conclusions

This contribution presents the case report of a young patient exhibiting relapsing MRI negative myelitis, diagnosed as MOGAD. MOGAD myelitis with normal spinal cord MRI has been reported in up to 10% of patients acutely and represent a major diagnostic challenge for clinicians [6–8]. However, this case report is unique by the involvement of relapsing isolated myelitis. The patient exhibited no symptoms other than myelitis over the 5-year follow-up period, and no symptoms or signs of visual or brainstem disturbances were apparent. Both visual and brainstem auditory evoked potentials and optical coherence tomography were normal. Most cases of MRI-negative MOGAD myelitis have been diagnosed in the light of other symptoms and signs of MOGAD, such as visual or brainstem manifestations [6]. The diagnosis of MOGAD was confirmed by repeated positivity of MOG-IgG in different labs (which suggested the high level of certainty). The titers presented herein were low, which is related to the well-known fact, that the MOG-IgG titers are significantly lower during remission comparing to the relapse titers [9].

Besides anti-MOG antibodies, the anti-AQP4 and anti-glial fibrillary acidic protein (GFAP) antibodies could be useful biomarkers in patients exhibiting similar symptoms reported herein, however the frequency of normal MRI in AQP4-IgG + myelitis is unknown. The myelitis associated with GFAP-IgG is different from both MOGAD and AQP4-IgG + myelitis because the symptoms presentation is typically slower (over weeks to months) and extra myelitis CNS symptoms are common accompaniments (meningo-encephalo-myelitis) [10]. Autoimmune myelitis with negative spinal cord MRI should be differentiated from patients with spinal cord infarction that may initially present with a normal spine MRI in approximately 25% of cases but presenting as an hyperacute spinal cord dysfunction [11], and patients with hereditary myelopathies that can have normal spinal cord MRI despite slowly progressive spinal cord dysfunction (e.g., adrenoleukodystrophy). Thus, a comprehensive evaluation of the clinical manifestations (e.g., timing of spinal cord dysfunction onset, CSF findings) should be performed to determine if MOG-IgG testing is appropriate. MOG-IgG detection in patients with hyperacute or slowly progressive spinal cord dysfunction over months should raise the suspicion of a false positive result as these clinical presentation are very uncommon in MOGAD [6]. The risk of false positive results is higher when MOG-IgG is tested by fixed rather than live cell-based assay.

Evoked potentials (EPs) are often neglected in the diagnostic workup of patients with demyelinating diseases (MS or NMOSD). Given its high sensitivity to subclinical lesions and relatively high specificity, MRI has largely replaced EPs in patients presenting with symptoms indicative of demyelinating disorders. However, the capacity of EPs to detect even those subclinical lesions of the main pathways that may not be well-explored in routine MRI assessments (e.g. optic nerve and/or spinal cord lesions), has been demonstrated [12]. The case report herein provides further support for the use of EPs in the evaluation of patients suspected of demyelinating diseases. In some cases, EPs may well prove more sensitive than MRI, although MRI still remains the most powerful tool in the diagnosis and classification of demyelinating disorders, in the study of their pathophysiology, and in following clinical courses and response to therapy. The high-resolution imaging of micro-anatomical depiction would offer potential benefits for patient treatment in terms of diagnostics and therapy monitoring. 7-T images provide a better anatomical visualization of tiny details and a better demarcation of gray and white matter, better spatial resolution, detection of anatomical and pathological features and it improves by 50% spinal cord MS lesion detection [13]. The use of ultra-high-field 7-T MRI is gaining growing popularity in investigating disease mechanisms in patients with demyelinating disease. The reasons why MOGAD attacks may rarely occur with negative MRI findings are incompletely understood. A predominant functional vs. structural damage in MOGAD compared to other myelitis (e.g., AQP4-IgG+, MS) might be an explanation. This is also in line with the tendency of MOGAD MRI lesions to resolve after the acute attacks, and the overall favorable outcome reported in MOGAD patients [14–16].

From the treatment perspective, the response to acute MOGAD relapses should be a high dose of steroids or, eventually, plasma exchange, while long-term treatment with immunosuppressive agents (azathioprine, mycophenolate mofetil, or anti-CD20 antibodies) should be considered [5]. The therapeutic approach herein followed these recommendations, and the patient appears to have been clinically stable for a year and a half.

Before the final diagnosis was established, CISP had been considered as a possible explanation for the patient’s clinical status. This was based on clinical symptoms and signs resembling peripheral nerve lesion (deep-tendon reflexes were absent and the plantar reflex was negative), relapsing-remitting course, an absence of relevant brain or spine MRI changes, cerebrospinal fluid albumino-cytological dissociation and response to corticosteroid therapy. None of the MRI examinations, however, confirmed any associated sacral nerve roots changes, which made, together with the sphincter dysfunction, a diagnosis of CISP highly improbable [3, 4].

MOGAD is one of the conditions which should be considered in MRI-negative myelitis. The diagnosis, however, may prove difficult, especially if the patient is not exhibiting other neurological symptoms of MOGAD. Conus or epiconus involvement is common in MOGAD; the patient reported herein exhibited incomplete rostral epiconus symptoms which, together with somatosensory evoked potential abnormalities, led to the diagnosis.

## Data Availability

The data presented in this study are available on request from the corresponding author.

## Abbreviations

MOG: Myelin-oligodendrocyte glycoprotein
MOGAD: MOG-IgG-associated disorder
MS: Multiple sclerosis
NMOSD: Neuromyelitis optica spectrum disorder
MRI: Magnetic resonance imaging
CRP: C-reactive protein
CSF: Cerebrospinal fluid
NCS/EMG: Nerve conduction studies and needle electromyography
CISP: Chronic immune sensory polyradiculopathy
AQP4: Anti-aquaporin-4
GFAP: Glial fibrillary acidic protein
Eps: Evoked potentials
GM: Gut microbiota

## References

[1] Banks SA, Morris PP, Chen JJ, Pittock SJ, Sechi E, Kunchok A (2021). Brainstem and cerebellar involvement in MOG-IgG-associated disorder versus aquaporin-4-IgG and MS. J Neurol Neurosurg Psychiatry.

[2] Dubey D, Pittock SJ, Krecke KN, Morris PP, Sechi E, Zalewski NL (2019). Clinical, Radiologic, and Prognostic Features of Myelitis Associated With Myelin Oligodendrocyte Glycoprotein Autoantibody. JAMA Neurol.

[3] Clerici AM, Nobile-Orazio E, Mauri M, Squellati FS, Bono GG (2017). Utility of somatosensory evoked potentials in the assessment of response to IVIG in a long-lasting case of chronic immune sensory polyradiculopathy. BMC Neurol.

[4] Van den Bergh PYK, Hadden RDM, Bouche P, Cornblath DR, Hahn A, Illa I (2010). European Federation of Neurological Societies/Peripheral Nerve Society guideline on management of chronic inflammatory demyelinating polyradiculoneuropathy: report of a joint task force of the European Federation of Neurological Societies and the Peripheral Nerve Society - first revision. Eur J Neurol.

[5] Whittam DH, Karthikeayan V, Gibbons E, Kneen R, Chandratre S, Ciccarelli O (2020). Treatment of MOG antibody associated disorders: results of an international survey. J Neurol.

[6] Sechi E, Krecke KN, Pittock SJ, Dubey D, Lopez-Chiriboga AS, Kunchok A (2021). Frequency and characteristics of MRI-negative myelitis associated with MOG autoantibodies. Mult Scler.

[7] Macaron G, Ontaneda D (2020). MOG-related disorders: A new cause of imaging-negative myelitis?. Mult Scler.

[8] Grangeon L, Hébant B, Guillaume M, Ahtoy P, Lefaucheur R (2020). Myelitis with normal spinal cord MRI: don’t forget anti-MOG antibodies disease!. Acta Neurol Belg.

[9] Jarius S, Ruprecht K, Kleiter I, Borisow N, Asgari N, Pitarokoili K (2016). MOG-IgG in NMO and related disorders: a multicenter study of 50 patients. Part 1: Frequency, syndrome specificity, influence of disease activity, long-term course, association with AQP4-IgG, and origin. J Neuroinflammation.

[10] Sechi E, Morris PP, McKeon A, Pittock SJ, Hinson SR, Weinshenker BG (2019). Glial fibrillary acidic protein IgG related myelitis: characterisation and comparison with aquaporin-4-IgG myelitis. J Neurol Neurosurg Psychiatry.

[11] Zalewski NL, Rabinstein AA, Krecke KN, Brown RD, Wijdicks EFM, Weinshenker BG (2019). Characteristics of Spontaneous Spinal Cord Infarction and Proposed Diagnostic Criteria. JAMA Neurol.

[12] Hardmeier M, Leocani L, Fuhr P (2017). A new role for evoked potentials in MS? Repurposing evoked potentials as biomarkers for clinical trials in MS. Mult Scler.

[13] Dula AN, Pawate S, Dortch RD, Barry RL, George-Durrett KM, Lyttle BD (2016). Magnetic resonance imaging of the cervical spinal cord in multiple sclerosis at 7T. Mult Scler.

[14] Sechi E, Krecke KN, Messina SA, Buciuc M, Pittock SJ, Chen JJ (2021). Comparison of MRI Lesion Evolution in Different Central Nervous System Demyelinating Disorders. Neurology.

[15] Cobo-Calvo A, Ruiz A, Maillart E, Audoin B, Zephir H, Bourre B (2018). Clinical spectrum and prognostic value of CNS MOG autoimmunity in adults: The MOGADOR study. Neurology.

[16] Lopez-Chiriboga AS, Sechi E, Buciuc M, Chen JJ, Pittock SJ, Lucchinetti CF (2020). Long-term Outcomes in Patients With Myelin Oligodendrocyte Glycoprotein Immunoglobulin G-Associated Disorder. JAMA Neurol.

